# The association of sensory phenotype and concomitant mood, sleep and functional impairment with the outcome of carpal tunnel surgery

**DOI:** 10.1186/s12891-021-04832-2

**Published:** 2021-11-17

**Authors:** Donna L. Kennedy, Deborah Ridout, Ladislava Lysakova, Jan Vollert, Caroline M. Alexander, Andrew S. C. Rice

**Affiliations:** 1 Pain Research, Department of Surgery and Cancer, Faculty of Medicine, Chelsea & Westminster Hospital Campus, Imperial College London, 369 Fulham Rd, London, SW10 9NH UK; 2 Therapies Department, Imperial College Healthcare NHS Trust, London, UK; 3 Population, Policy and Practice Programme, University College London Great Ormond St Institute of Child Health, London, UK; 4 Department of Plastic and Reconstructive Surgery, Imperial College Healthcare NHS Trust, London, UK; 5 Division of Neurological Pain Research and Therapy, Department of Neurology, University Hospital of Schleswig-Holstein, Campus Kiel, Germany; 6 Department of Anaesthesiology, Intensive Care and Pain Medicine, University Hospital Muenster, Muenster, Germany; 7 Neurophysiology, Mannheim Center of Translational Neuroscience (MCTN), Medical Faculty Mannheim, Heidelberg University, Heidelberg, Germany; 8 MSk Lab, Department of Surgery and Cancer, Imperial College London, London, UK

**Keywords:** Anxiety, Carpal tunnel surgery, Catastrophizing, Insomnia, Neuropathic pain, Phenotype, Quantitative sensory testing (QST)

## Abstract

**Background:**

Up to 25% of people who have had carpal tunnel release surgery (CTR) fail to report improvement; however, evidence for prognostic indicators in this surgical cohort is limited. To identify candidate prognostic factors, this study investigated the association of quantitative sensory testing (QST) derived sensory phenotype and attendant impairment with patient-reported surgical outcome.

**Methods:**

With ethical approval and informed consent, this prospective observational longitudinal study recruited patients from two London hospitals. Multimodal phenotyping measures including quantitative sensory testing (QST), pain parameters, insomnia, pain-related worry, mood and function, were evaluated prior to; and at 3- and 6-months post-surgery. Pain in median nerve distribution with electrophysiologically confirmed conduction delay and DN4 score ≥ 4 was defined as neuropathic. Primary outcome was patient-rated change at 6 months, dichotomised as poor outcome; “worse” or “no change” and good outcome; “slightly better”, “much better” or “completely cured”.

**Results:**

Seventy-six patients participated. Prior to surgery, substantial heterogeneity in established categories of somatosensory function was observed with 21% of participants categorised as having a healthy sensory phenotype; 29% with thermal hyperalgesia; 32% mechanical hyperalgesia and 18% sensory loss. Seventy six percent of participants were classified as having neuropathic pain, 33% with high levels of pain related worry and 64% with clinical insomnia. Observed differences in pain, sleep impairment, psychological factors and function, between sensory phenotypic groups, was not significant. At 3- and 6-months post-surgery there was significant improvement in all phenotyping measures with a moderate to large effect size. Thermal and mechanical measures of somatosensation improved (*p* < 0.001), as did functional ability (*p* < 0.001). Symptom severity diminished (*p* < 0.001), as did pain-related worry (*p* < 0.001), anxiety (*p* = 0.02) and insomnia (*p* < 0.001). Patient-rated surgical outcome was good in 92% of the cohort, poor in 8%. Baseline sensory phenotype category was not associated with surgical outcome however pain-related worry, anxiety and functional interference were significantly associated with outcome (*p* ≤ 0.05).

**Conclusion:**

In patients undergoing carpal tunnel surgery, pain-related worry, anxiety and pain functional interference are candidate prognostic outcome factors and require further elucidation.

## Background

Carpal tunnel syndrome (CTS), compression of the median nerve at the wrist, is the most prevalent of the entrapment neuropathies [1]. Prevalence estimates vary based on diagnostic criteria; however, it is estimated that one in 10 people will develop carpal tunnel syndrome at some point [2]. CTS symptoms include pain, paraesthesiae and/or numbness in the median nerve distribution of the hand and weakness of the thenar muscles. Symptoms impair performance of daily activities and adversely affect quality of life. Median nerve decompression surgery, or carpal tunnel release (CTR), is an efficacious treatment for this potentially debilitating condition and is the most commonly performed procedure in the hand [3, 4]. However, carpal tunnel surgery is not without risk of adverse events [5, 6] and significantly, up to 25% of patients fail to report improvement following surgery [7, 8].

Clearly, it would be advantageous if clinicians, prior to surgery, could anticipate treatment response for a given patient. This might underpin a personalised medicine approach, guiding patient stratification, modification in care pathways or enabling the use of ‘prehabilitation’ approaches to surgery preparedness [9]. However, at present, there is limited evidence for prognostic factors associated with CTR outcome. The severity of electrophysiologically assessed nerve conduction delay has been investigated extensively, but findings from observational studies are contradictory and inconclusive [10–20]. Studies employing a prognostic factor design [21] have identified that greater functional impairment [22], increased utilisation of health care resources, a greater number of comorbid health conditions and higher levels of anxiety [23] prior to surgery are prognostic factors associated with poorer surgical outcome. Consistent with these findings, a review of the CTR outcome literature concluded that poorer general health and comorbid conditions including diabetes, alcohol consumption and smoking were associated with a worse surgical prognosis [19].

While pain is prevalent in CTS [2] and is neuropathic in nature in up to 80% of patients [24, 25], the mechanistic nature of pain in CTS has not been widely studied nor fully elucidated [26, 27]. It is recognised that for patients with neuropathy, pain is heterogeneous, manifesting diversely across those with a common clinical condition [28]. An individual’s profile of sensory symptoms and signs, or sensory phenotype [29], is thought to reflect this inter-individual heterogeneity in underlying pathophysiology of pain pathways and mechanisms in neuropathic pain conditions [30–33].

It is acknowledged that neuropathic pain, caused by a lesion or disease of the somatosensory nervous system [34, 35] poses a considerable, multidimensional burden for patients [36]. Secondarily, consensus group evaluation guidelines are underpinned by the biopsychosocial model of pain, consistent with the International Classification of Functioning, Disability and Health [37]. Accordingly, in addition to pain parameters, the domains of mood, sleep and function are integral elements of a patient-centred evaluation [38, 39] and resonate with patient priorities [40].

Sensory phenotype has previously been described in patients with carpal tunnel syndrome, demonstrating heterogeneity in small and large median nerve fibre somatosensory dysfunction [25, 41], however it is not clear if sensory phenotype is associated with the outcome of carpal tunnel surgery. Furthermore, it is not known if attendant burden in the domains of pain, mood, sleep interference and functional impairment differs between sensory phenotypic groups, or if these domains are prognostic factors associated with surgical outcome. Identifying intra-individual factors associated with patient-reported symptom severity and surgical outcome may inform stratified care for patients with carpal tunnel syndrome, better guiding treatment decisions and thereby improving outcomes.

We aimed to investigate the association of sensory phenotype and concomitant pain parameters, psychological factors, sleep restriction and functional impairment with outcome at 6 months post carpal tunnel surgery in order to identify candidate prognostic factors for future investigation. Secondarily, we aimed to explore heterogeneity in sensory phenotype and associated comorbidity in the domains of pain, mood, sleep and function in order to establish whether symptom clusters are identifiable within the population of patients with carpal tunnel syndrome. The hypothesis being, in patients with carpal tunnel syndrome, there is a constellation of neuropathic pain associated clinical features which predispose this phenotypic group to symptoms which do not improve or worsen following surgery.

## Methods

Ethical approval was granted by the Camberwell St Giles National Research Ethics Committee (14/LO/1436) on 29 August 2014 for a prospective, longitudinal observational study. Two patient-collaborators (SP; AT), previewed the study measures and procedures and edited study documents for clarity and acceptability. Sequential adult patients listed for open carpal tunnel decompression surgery at two London National Health Service (NHS) hospitals were recruited by poster and in person at their hospital clinic appointment and by post. A comparable surgical technique was used at the two recruitment sites; open decompression with a median nerve block under tourniquet control. Incisions were closed with non-absorbable sutures. Participants were not paid for study participation however travel was reimbursed.

### Participation criteria

Exclusion criteria were significant cognitive dysfunction, patient-defined lack of English language adequate for completing study questionnaires and participating in psychophysical testing, a history of potentially confounding conditions (rheumatoid arthritis, renal failure, peripheral neuropathy of any origin other than CTS), steroid injection of the study limb within the previous 4 weeks or previous carpal tunnel surgical release in the study hand, anatomic abnormalities of the wrist or hand, median nerve injury or compression secondary to traumatic injury and pregnancy.

### Sample size

Participants were stratified by sensory phenotype using the German Research Network on Neuropathic Pain (DFNS) quantitative sensory testing (QST) protocol [42] and algorithm [43]. Published age and sex stratified QST reference data for the hand pertain to the dorsal, radial nerve innervated hand [44] and are not generalisable to the volar, median nerve innervated hand [25]. Therefore, to identify sensory dysfunction in this CTS cohort, sample size was calculated based on an independent samples t-test to determine a difference in cold pain threshold (CPT), as evaluated with the DFNS QST protocol, for patients with carpal tunnel syndrome tested at the volar middle finger (mean 17.3; ±5.9 °C) and healthy controls (mean 13.4 ± 7.5 °C) [45]. Control data was generated from a convenience sample of participants in a previously reported healthy volunteer quantitative sensory testing study [46]. Of the thirteen QST measures in the DFNS battery, CPT was chosen as a measure of importance because in other cohorts, cold pain sensitivity has been demonstrated to be associated with pain and disability outcomes [47] and surgical outcome [48]. A minimum sample size of 94 (47 CTS participants and 47 healthy controls) was required to achieve a power of 80% and a level of significance of 5% (two sided), for detecting a difference of a similar size for QST.

### Surgical outcome

The measure used to categorize surgical outcome as good or poor (binary outcome) was a patient-reported global rating of change (PGRC) at 6 months post-surgery. The PGRC is a 5-point ordinal scale whereby 1 = worse; 2 = unchanged; 3 = slightly better; 4 = much better and 5 = completed cured [7, 18, 49, 50] with a grade of 3 or above interpreted as a good outcome or treatment success. Where investigators [7, 49] have defined treatment success as 4 or above using the same ordinal scale, they note that their patients are selected for surgery based on a good prognosis, therefore their findings are less generalizable to the wider population of patients. However, in a comparable pragmatic prospective cohort including participants with multiple comorbidities, a grade of 3 (slightly better) was similarly identified as representative of treatment success [50]. Other surgical outcome measures included patient rated symptom severity and functional impairment evaluated with the Boston Carpal Tunnel Questionnaire (BCTQ) [51] and post-surgical scar pain and interference [52]. Participants sealed completed outcome measures in pre-labeled, coded envelopes which were secured in the written case report form thereby blinding the investigator (D.K.) until participants completed the trial.

### Procedure

Median nerve somatosensory function, pain parameters, psychological state and quality of life measures were evaluated prior to and at 3- and 6-months post-surgery. Baseline measures were completed within 6 weeks prior to surgery; 3- and 6-months post- surgery assessments were completed within ±21 days. Where participants failed to attend a 3- or 6-month assessment, outcome questionnaires were posted. At baseline, demographic data and medical history was recorded. Clinical history taking utilised the Sangha Comorbidity Questionnaire, a patient-reported tool validated for quantifying comorbid conditions and their impact on function, thereby enabling the comparison of general health between participants [53]. Comorbidity scores range from 0 to 45, with higher scores implying poorer general health and functional impairment. All tests and questionnaires were delivered in the same order across the participants, across visits.

### Definition of neuropathic pain

Nerve conduction studies (NCS) were performed by the respective hospital neurophysiology departments and severity graded according to Bland [54] criteria. Pain was categorised as neuropathic where there was neurophysiologic confirmation of median nerve conduction delay; a score of ≥4 on the Douleur Neuropathique 4 questions (DN4) *and* where pain was present in the median nerve distribution as reported on a participant completed pain map [34, 38, 55]. Post-surgery this two-stage triage was repeated however, *in lieu* of repeat electrophysiological testing, two or more abnormal QST findings indicative of loss of sensory function was taken as a confirmatory diagnostic test for neuropathic pain.

### Multimodal Phenotyping measures

Relevant phenotypic characteristics and measures were derived from the Initiative on Methods, Measurement, and Pain Assessment in Clinical Trials (*IMMPACT*) consensus guidelines for patient phenotyping in clinical trials of pain treatments [56], commensurate with consensus guidelines for the assessment of patients with neuropathic pain [38]. Phenotypic characteristics of interest included quantitative sensory testing (QST) derived sensory phenotype, pain parameters, psychological factors including catastrophic thinking in relation to pain, anxiety and depression, sleep restriction and functional impairment.

### Sensory phenotype

Somatosensory function was evaluated according to the German Research Network on Neuropathic Pain (DFNS) quantitative sensory testing (QST) protocol [42]; indepth methods have been previously reported elsewhere [25]. All equipment was calibrated prior to testing. In brief, thermal detection thresholds, thermal pain thresholds and thermal sensory limen were evaluated with a Somedic MSA thermal stimulator (Sweden) with an 18 mm^2^ metal Somedic thermode. Mechanical detection threshold was tested using glass monofilaments (Optihair2-Set, Marstock Nervtest, Germany) with bending forces between 0.25 and 512 mN. Mechanical pain threshold, mechanical pain sensitivity and wind-up ratio were evaluated using blunt probes with forces ranging from 8 to 512 mN (pinprick stimulator, MRC, Heidelberg, Germany). Dynamic mechanical allodynia was tested with a cotton wisp, a cotton bud (Q-Tip) and a standardised brush designed to produce minimum friction (Somedic, Sweden). Vibration detection threshold testing used a Rydel–Seiffer graded tuning fork (64 Hz, 8/8 scale). Pressure pain threshold was tested with a pressure algometer (FDN100, Wagner Instruments, Greenwich, CT, USA) with a surface area of 1 cm^2^ and by applying pressure at a rate of 1 kg/cm^2^ per second. Pressure pain threshold was tested at the thenar eminence, all other tests were performed at the volar distal phalanx of the middle finger.

**Fig. 1:**
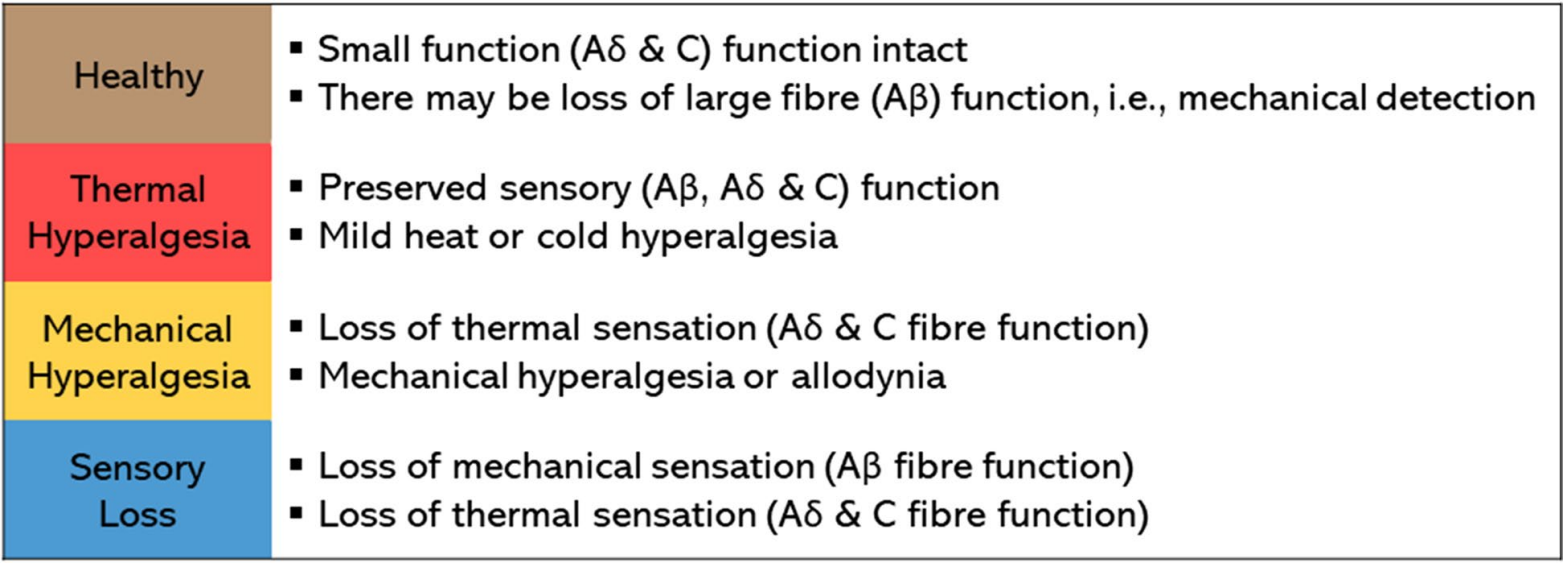
Quantitative sensory testing (QST) derived sensory phenotypes [43] and associated sensory impairment characteristics

Using the DFNS algorithm [43], participants were categorised as having a “healthy” sensory phenotype or stratified to a thermal hyperalgesia, mechanical hyperalgesia or sensory loss phenotype (Fig. 1) [57]. In the context of testing patients with CTS, a “healthy” sensory phenotype suggests that sensory function is not characterised by small fibre dysfunction, as would be consistent with neuropathy.

### Pain parameters

Pain symptoms, signs and descriptors were assessed with the Douleur Neuropathique 4 questions (DN4) [58]. The DN4 consists of seven symptom questions and three sensory examination measures; a score of > 4 is considered diagnostic of neuropathic pain. Pain dimensions were assessed with the Neuropathic Pain Symptom Inventory (NPSI) [59], a validated patient-completed inventory for evaluating the nature of neuropathic pain, including spontaneous, paroxysmal and evoked pain and paraesthesia/dysesthesia. Total NPSI scores range from 0 to 100 with greater scores implying more severe symptom severity; item scores of 1–3 indicate mild pain severity, 4–6 moderate and 7–10 severe [60]. Pain severity and interference was assessed with the validated [61] Brief Pain Inventory (BPI) [62]. The BPI is a two-part questionnaire whereby a Pain Severity Score (PSS) is calculated as the mean of four questions quantifying present pain and the least, worst, and average pain over the last week. Pain is rated on an 11-point scale ranging from 0 (no pain) to 10 (pain as bad as you can imagine). There are no universally accepted cut-points for interpreting pain scales however in patients with chronic musculoskeletal pain, ratings of 1–3 out of 10 are suggested to correspond with mild pain, 4–6 with moderate pain and 7–10 severe pain [63, 64]. Pain severity and frequency was assessed with the Boston Carpal Tunnel Questionnaire (BCTQ) Symptom Severity Scale (SSS) [51], a CTS specific, patient-completed questionnaire which is well validated, reliable and responsive [65, 66]. Eleven symptoms are rated on a 5-point scale with lower scores implying milder symptoms.

### Psychological factors

Pain-related worry was evaluated with the Pain Catastrophizing Scale (PCS) [67]. Pain catastrophising is defined as exaggerated, persistent thought related to painful experiences coupled with a perceived inability to cope with such experiences [67, 68]. However, at present, questions have arisen as to the validity of assessing pain catastrophizing based on self-report measures; it is proposed the construct being evaluated with self-report measures is more appropriately described as ‘pain-related worry’ [69]. The PCS yields a total score (range 0–52) and three subscale scores assessing rumination (range 0–16), magnification (range 0–12) and helplessness (range 0–24). A PCS score of 30 or greater is described as indicative of clinically relevant pain-related worry. Mood was assessed with the Depression, Anxiety and Positive Outlook Scale (DAPOS) [70] an 11-item questionnaire. The DAPOS was developed and validated specifically for use in patients with chronic pain. The DAPOS has 3 independent scales; depression, anxiety and positive outlook. Scores for the subscales range from 5 to 25 for depression, 3 to 15 for anxiety, and 3 to 15 for positive outlook. There is no total score for the DAPOS nor a defined score for clinically relevant depression or anxiety [71].

### Sleep

Sleep interference was evaluated with the Insomnia Severity Index (ISI) [72], a 7 item measure (score range 0–28) that quantifies sleep disturbance and the impact of insomnia on function and quality of life. Scores of 7 or less are interpreted as suggesting no clinically significant insomnia; 8–14 as subthreshold insomnia; 15–21 as clinical insomnia of moderate severity and 22–28 as severe clinical insomnia.

### Functional impairment

Functional impairment was evaluated with the patient-completed Boston Carpal Tunnel Questionnaire (BCTQ) [51] 8 item Functional Status Scale (FSS). Items are rated on a 5-point scale with lower scores imply milder symptoms and less functional impairment. Additionally, pain interference was assessed with the Brief Pain Inventory (Short Form) (BPI-SF) Interference Scale [62]. Seven BPI-SF items are scored on an 11-point scale ranging from 0 (does not interfere) to 10 (completely interferes), quantifying pain interference in general activity, walking, work, relationships, mood, life enjoyment, and sleep and reported as the mean of the seven items.

### Statistical analysis

All continuous data were tested for normality of distribution. To categorise sensory phenotype, QST results were compared to control data generated from a convenience sample of healthy volunteers [46]. Z-scores were calculated (*z* = [value of participant - mean value of controls] / standard deviation of controls) [44]. Values outside the range of z x ± 1.96 were interpreted as abnormal, positive z scores denote a gain in function (hyperalgesia) whereas negative scores indicate a loss of function.

Patient characteristics and distribution of phenotyping measures were summarized using descriptive statistics. To identify differences in attendant burden (i.e., pain, mood, sleep impairment, functional impairment) between sensory phenotypic groups, differences in phenotyping measures were analysed with the non-parametric Kruskal-Wallis Test; comparison of pairs was conducted with the Mann-Whitney U Test (actual, not corrected *P* values reported).

Change in multimodal phenotyping measures across three time points (baseline; 3 months; 6 months) was investigated with one-way repeated measures analysis of variance (ANOVA) with Bonferroni correction and pairwise comparisons or the non-parametric Friedman test, as appropriate. Effect size was investigated with partial eta squared [73] and interpreted as 0.01 = small, .05 = medium and 0.14 = large [74].

The primary outcome, patient completed global rating of change (PGRC) at 6 months post-surgery was used to classify surgical outcome as a binary variable; a good versus poor outcome. A score of 3 or better was interpreted as a good outcome, or treatment success. Chi-square test for independence was used to investigate the relationship between sensory phenotype and patient-reported surgical outcome; significance is reported for the Pearson Chi-Square value or Fisher’s Exact Probability Test where cell counts were less than 5. Whereas the analysis plan was to explore the relationship between baseline multimodal phenotyping measures (pain parameters, psychological factors, sleep restriction and functional impairment) with carpal tunnel surgery outcome (PGRC) using multivariate analysis of variance, this was precluded by the small number of participants in the “poor surgical outcome” group. Therefore, the association of phenotyping measures and surgical outcome was investigated with the Spearman Rank Order Correlation Coefficients. Where correlation coefficients were statistically significant (*p* ≤ .05), the strength of relationship was interpreted as small *r* = .10 to .29; medium *r* = .30 to .49; large *r* = .50 to 1.0 [74].

## Results

**Fig. 2:**
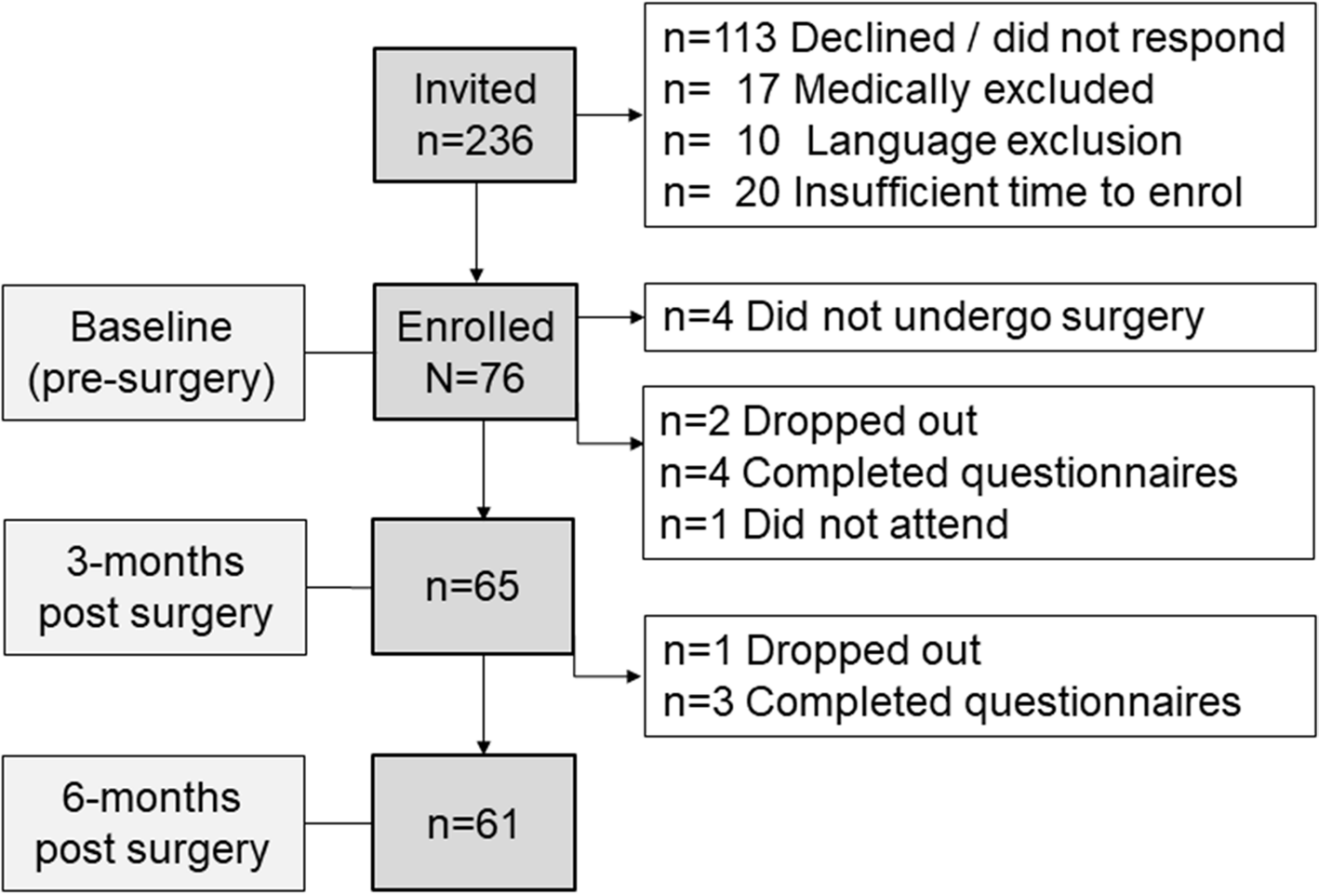
Study recruitment and enrolment

Seventy-six participants were enrolled between October 2014 and December 2016 and completed baseline study measures; however, 4 participants did not undergo surgery (one patient declined surgery and in 3 cases surgery was cancelled due to ongoing medical investigations) (Fig. 2). Demographic data and health parameters for the sample are reported in Table 1. The sample was comprised predominantly of white females who were currently employed and undergoing surgery on their dominant hand (*n* = 53; 70%). In the majority of participants (76%), severity of nerve compression was graded from moderately to extremely severe (very mild 4%; mild 21% moderately severe 29%; severe 18%; very severe 21%; extremely severe 3%) [54].

**Table 1:** Key demographic and health parameters

Age mean years (SD)	58.5 (13.5)
Female sex n (%)	65 (86)
Body mass index mean (SD)	28.8 (6.8)
Comorbidity score mean (SD)	5.1 (4.0)
Symptom duration mean months (SD)	52 (48)
Smoking history n (%)	
Never smoked	30 (39)
Previous smoker	35 (46)
Present smoker	11 (15)
Ethnicity n (%)	
White	48 (64)
Asian	11 (15)
Black	12 (16)
Mixed	4 (5)
Arab	1 (1)
Profession n (%)	
Manual; service trades	29 (38)
Administrative & technical	17 (22)
Professionals	30 (39)
Employment history n (%)	
Employed	42 (55)
Unemployed	12 (16)
Retired	22 (29)

Key: Standard deviation (SD)

### Sensory phenotype

**Fig. 3:**
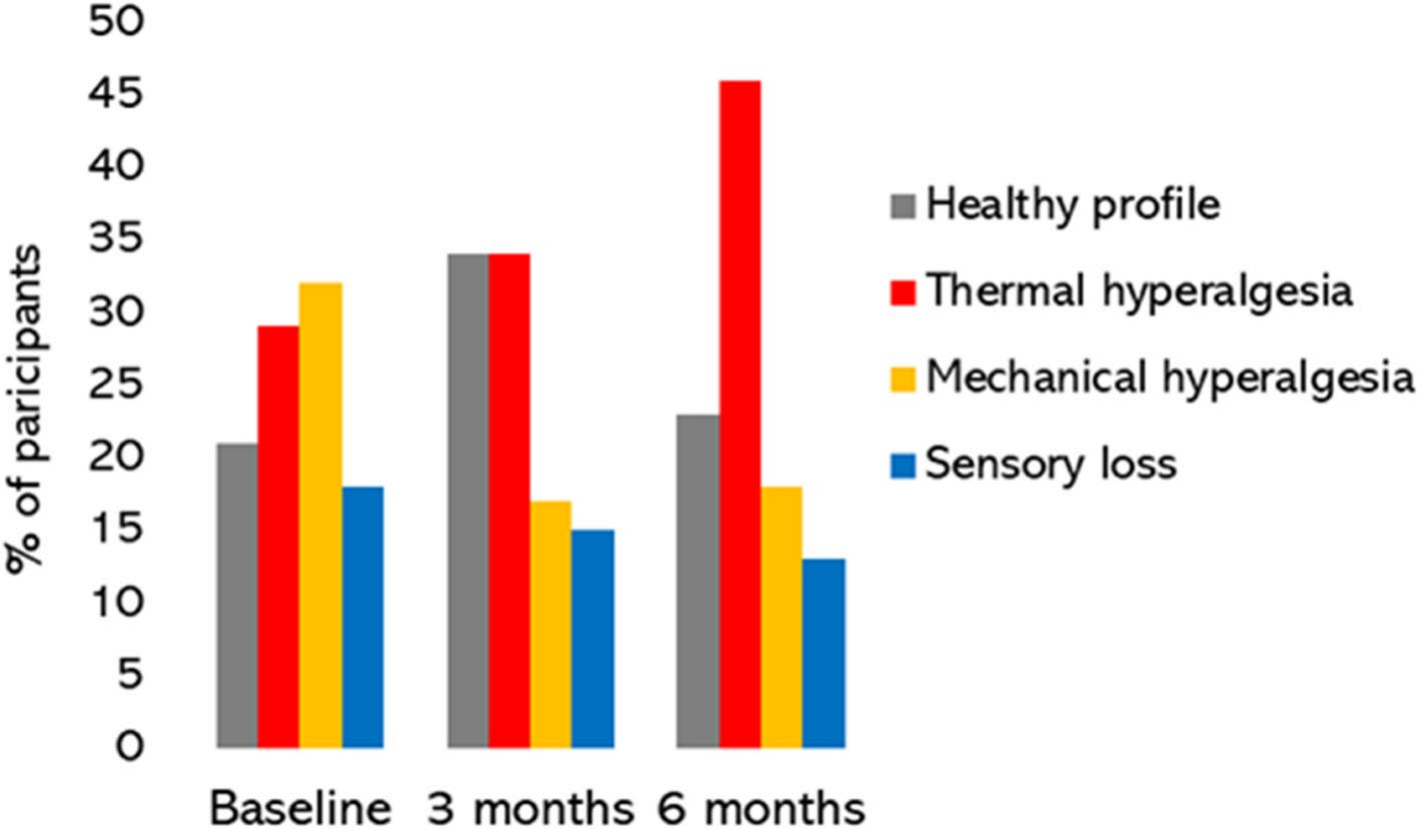
Quantitative sensory testing (QST) derived sensory phenotypes at baseline, 3- and 6-months post-surgery

To derive sensory phenotype, QST results were compared to control data from a previously reported convenience sample of 54 healthy volunteers [mean age 54.9 years (standard deviation 11.3); 38 (70%) female] [25]. At baseline, 16 (21%) of participants were defined as having a “healthy” sensory profile, 22 (29%) thermal hyperalgesia, 24 (32%) mechanical hyperalgesia and 14 (18%) sensory loss phenotype. Change in sensory phenotype from baseline to 3 and 6-month post-surgical assessments was statistically significant (*p* < .001) (Fig. 3).

**Fig. 4:**
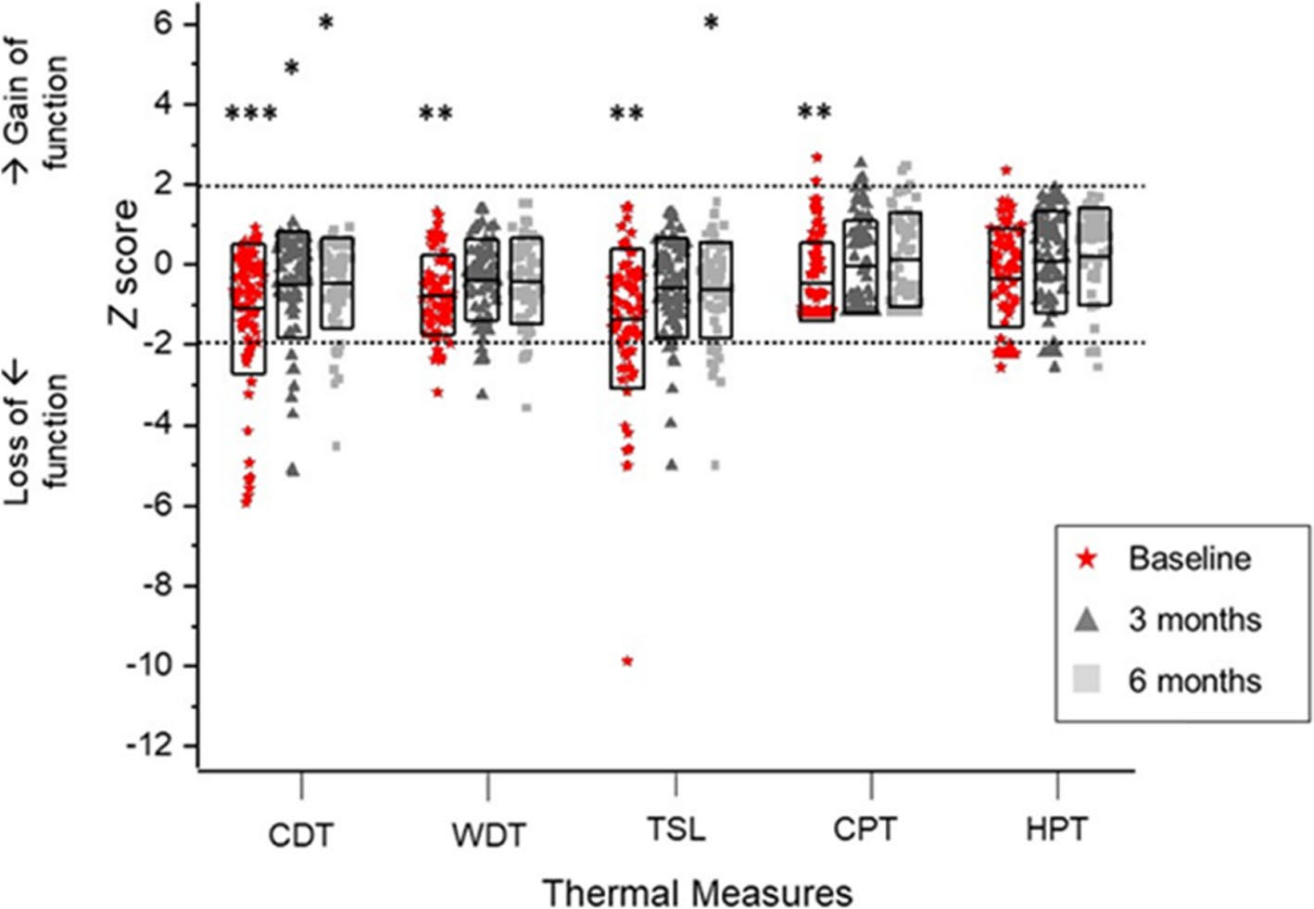
QST thermal measures comparing CTS participants and control data. Boxes represent the interquartile range, the centre line the median. The black upper dotted line represents + 1.96z, the bottom dotted line - 1.96z. Scores between the two are interpreted as normal, those above as gain of function and below as loss of function. Significance is denoted as * at the 0.05 probability level; ** at 0.01; *** at 0.001. Cold detection threshold (CDT); cold pain threshold (CPT); heat pain threshold (HPT); thermal sensory limen (TSL); warm detection threshold (WDT)

**Fig. 5:**
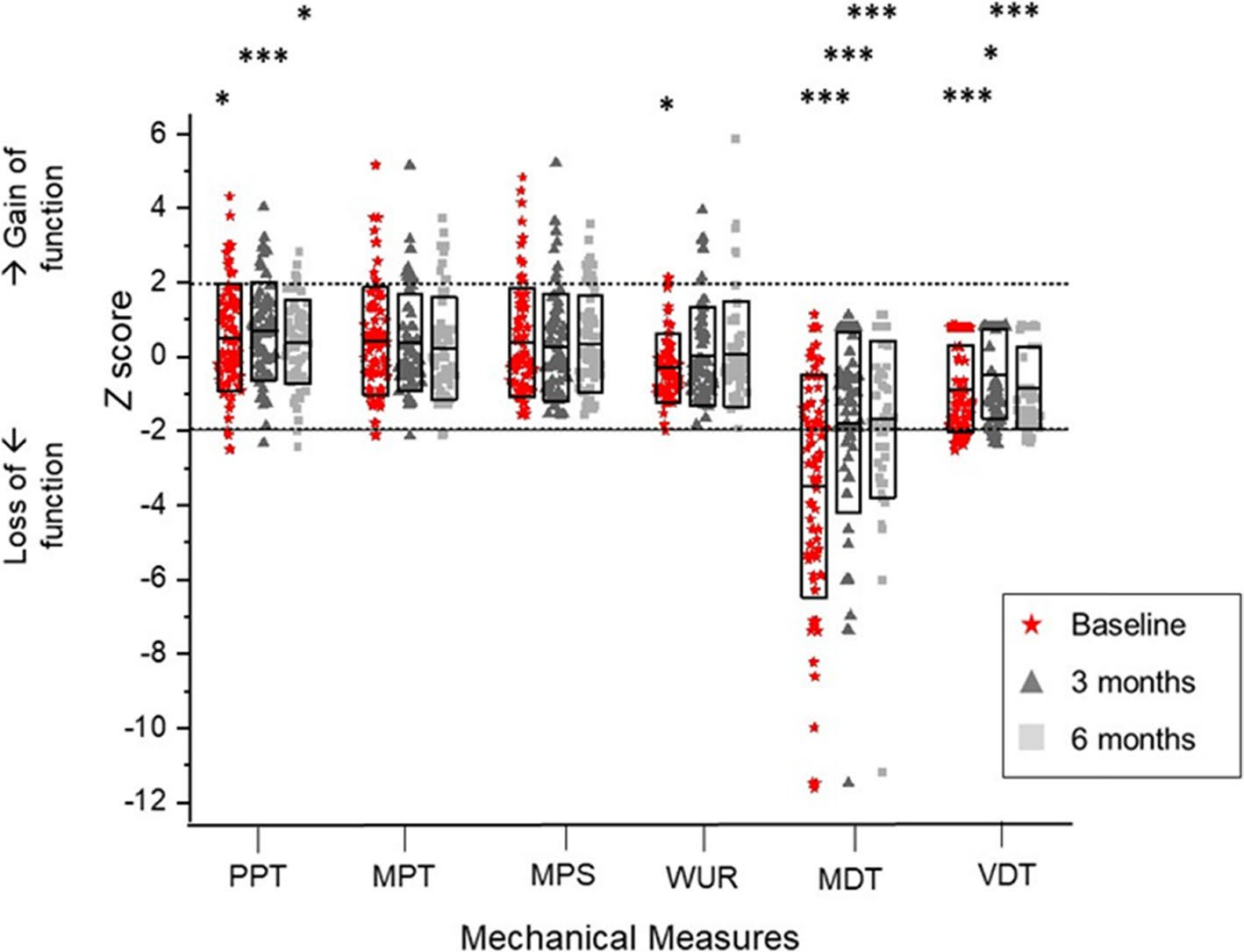
QST mechanical measures comparing CTS participants and controls. Boxes represent the interquartile range, the centre line the median. The black upper dotted line represents + 1.96z, the bottom dotted line - 1.96z. Scores between the two are interpreted as normal, those above as gain of function and below as loss of function. Significance is denoted as * at the 0.05 probability level; ** at 0.01; *** at 0.001. Mechanical detection threshold (MDT); mechanical pain sensitivity (MPS); mechanical pain threshold (MPT); pressure pain threshold (PPT); vibration detection threshold (VDT); wind-up ration (WUR)

QST results, priority to surgery, clearly illustrate this marked heterogeneity in somatosensory function. Loss of small fibre function (thermal detection and cold hyperalgesia) was observed in up to 25% of patients (Fig. 4); whereas paradoxical heat sensations, a pathological response, were rarely observed (pre-surgery 14%; 3 months post-surgery 17%; 6 months post-surgery 10%). Loss of large fibre function (mechanical detection threshold and vibration detection threshold) was the predominant sensory feature, identified in up to 60% of participants. In contrast, mechanical hyperalgesia, as evidenced by decreased mechanical pain threshold and increased mechanical pain sensitivity, was only observed in 16% of the cohort (Fig. 5). Finally, dynamic mechanical allodynia, a pathological sensory response, was not exhibited by CTS participants prior to or following surgery.

### Pain parameters

All pain parameters were normally distributed. At baseline, 76% of participants reported pain that was categorised as neuropathic in nature. Change in DN4 scores were statistically significant from baseline to 3 months and baseline to 6 months (*p* < .001) (Table 2**).** Of the 32 participants who continued to report pain at 6 months post-surgery, in 24 (38%) this was mild, 8 (13%) moderate and 3(5%) severe [63, 64].

**Table 2:** Change in DN4 and incidence of neuropathic pain

	Baseline (*n* = 76)	3 months (*n* = 65)	6 months (*n* = 61)
DN4 mean (sd)	5.39 (2.05)	1.81 (1.77)	1.29 (1.91)
Pain Category			
Pain free n (%)	7 (9)	25 (39)	29 (48)
Non-neuropathic n (%)	11 (15)	33 (51)	25 (41)
Neuropathic n (%)	58 (76)	7 (11)	7 (12)

Key: standard deviation (sd)

For all pain parameters, there was a significant effect for time (*p* < .001) and the magnitude of the effect size was large, demonstrating significant improvement in pain post-surgery. Differences in baseline pain parameters, between sensory phenotypic groups, were explored. In addition, baseline sensory phenotype was used to investigate if there were observable differences in pain trajectories between sensory phenotypic groups at 6 months post-surgery (Table 3). For all pain parameters, both pre-surgery and at 6 months post-surgery, burden is lowest in participants with a healthy sensory phenotype and highest in those with a sensory loss phenotype. While baseline between-group differences were not statistically significant (Kruskal-Wallis Test *p* > .05), differences at 6 months approached significance for the Symptom Severity Scale score and were significant for the NPSI (*p* = 0.04).

**Table 3:** Pain parameters at baseline & 6 months post-surgery by sensory phenotype

Baseline Sensory Phenotype	Symptom Severity Score		BPI Pain Severity Score		NPSI	
	baseline	6 months	baseline	6 months	baseline	6 months
healthy profile	2.87 (0.8)	1.36 (0.6)	3.25 (4)	0.75 (3.3)	31.5 (40.5)	0 (9.5)
thermal hyperalgesia	3.18 (1.3)	1.23 (0.4)	5.0 (5.3)	0.0 (0.4)	29.0 (47.0)	2 (4.0)
mechanical hyperalgesia	3.27 (1.2)	1.50 (1.6)	3.88 (4.7)	0.75 (4.5)	32.5 (38.5)	7 (29.0)
sensory loss	3.27 (1.8)	1.73 (1.2)	5.0 (4.2)	0.38 (2.6)	39 (35.25)	5.5 (27.3)
*p* =	0.39	0.08	0.42	0.11	0.43	**0.04**

Data reported with median (interquartile range)

Baseline sensory phenotype: healthy profile *n* = 16, 6 months *n* = 13; thermal hyperalgesia *n* = 22, baseline *n* = 20; mechanical hyperalgesia *n* = 24, 6 months *n* = 18; sensory loss *n* = 14, 6 months *n* = 10. Statistical significance reported for Kruskal-Wallis test

*NPSI* Neuropathic Pain Symptom Inventory, *SSS* Symptom Severity Scale score

### Pain-related worry

**Fig. 6:**
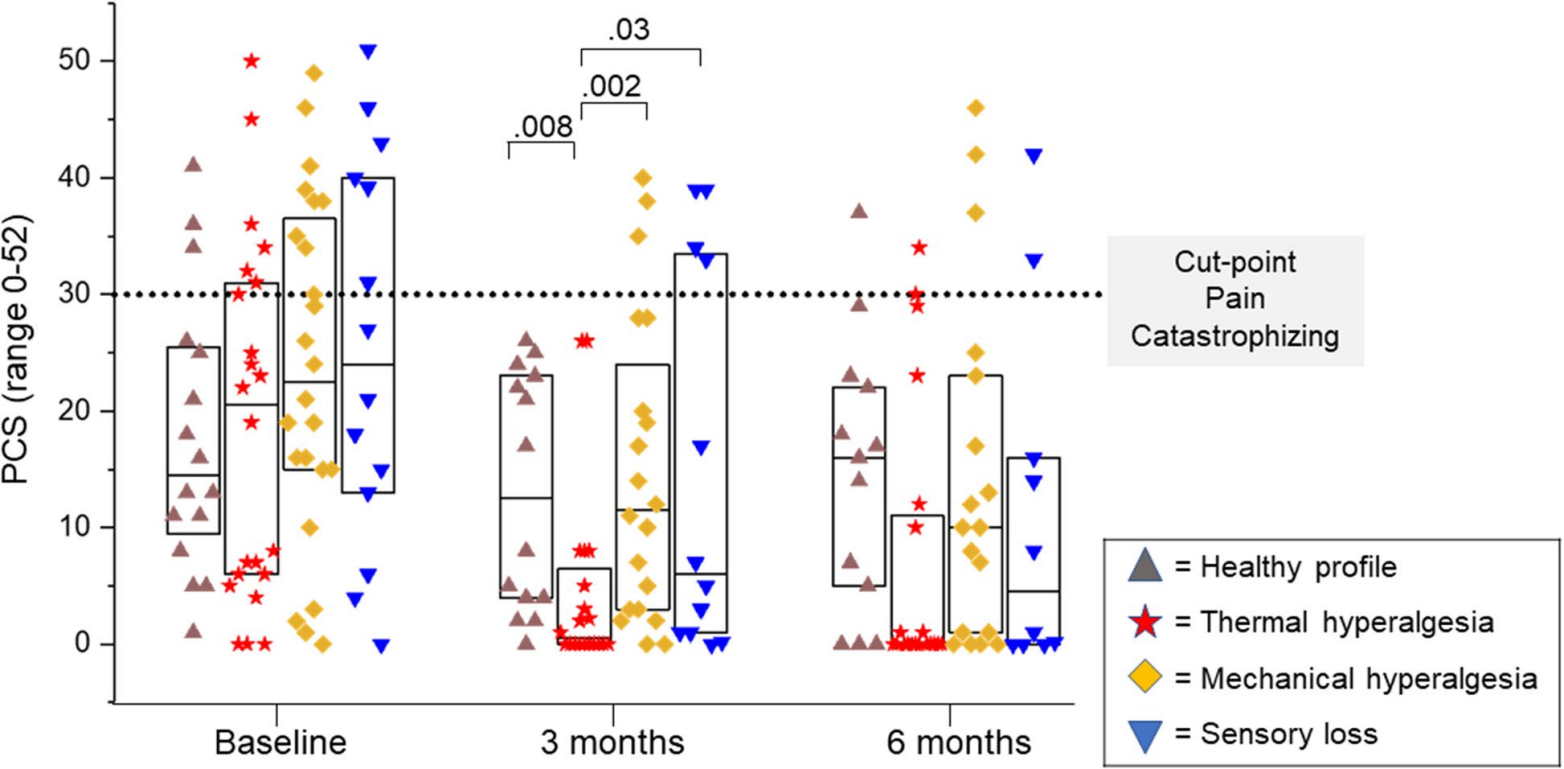
Pain Catastrophizing Scale (PCS). Differences in total Pain Catastrophizing Scale Score based on baseline phenotype group, at baseline, 3- and 6-months post-surgery. Box represents 25th and 75th percentiles, centre line the median. Statistically significant differences are reported in bold

At baseline, 25 (33%) of participants presented with clinically relevant pain-related worry based on Pain Catastrophizing Scale (PCS) scores; at 3 months this was reduced to 7 (11%) and at 6 months 8 (13%) (Fig. 6). Change in total PCS score from baseline (mean 20.08, standard deviation 13.36) to 3 months (mean 11.02, standard deviation 12.37) and baseline to 6 months (mean 10.36, standard deviation 12.39) was statistically significant (*p* < .001). Differences in the magnitude of pain-related worry between sensory phenotypic groups were explored prior to and post-surgery. At baseline, scores are lower in those with a healthy sensory phenotype and highest in those with a sensory loss phenotype, however group differences are not significant (*p* = 0.43). While between group differences were statistically significant at 3 months post-surgery (*p* = .007); differences at 6 months post-surgery were not (*p* = 0.12).

### Mood

The DAPOS scales for depression, anxiety and positive outlook were analysed separately, there is no “total” DAPOS score. No change in depression scores were observed post-surgery for the sample (baseline median 6 [IQR 3]; 6 months median 5 [IQR 2] (*p* = .42). In contrast, following surgery, a significant decrease in anxiety and increase in positive outlook was observed. Baseline anxiety score (median 4 [IQR 4]) decreased at 3 months (median 3.5 [IQR 2]) (*p* = .02) and 6 months (median 4 [IQR 2]) (*p* = .04) while positive outlook scores increased from baseline (median 11 [IQR 5]) to 3 months (median 12 [IQR 5]) and 6 months (median 11 [IQR 5]) (*p* ≤ .02).

### Sleep impairment

**Fig. 7:**
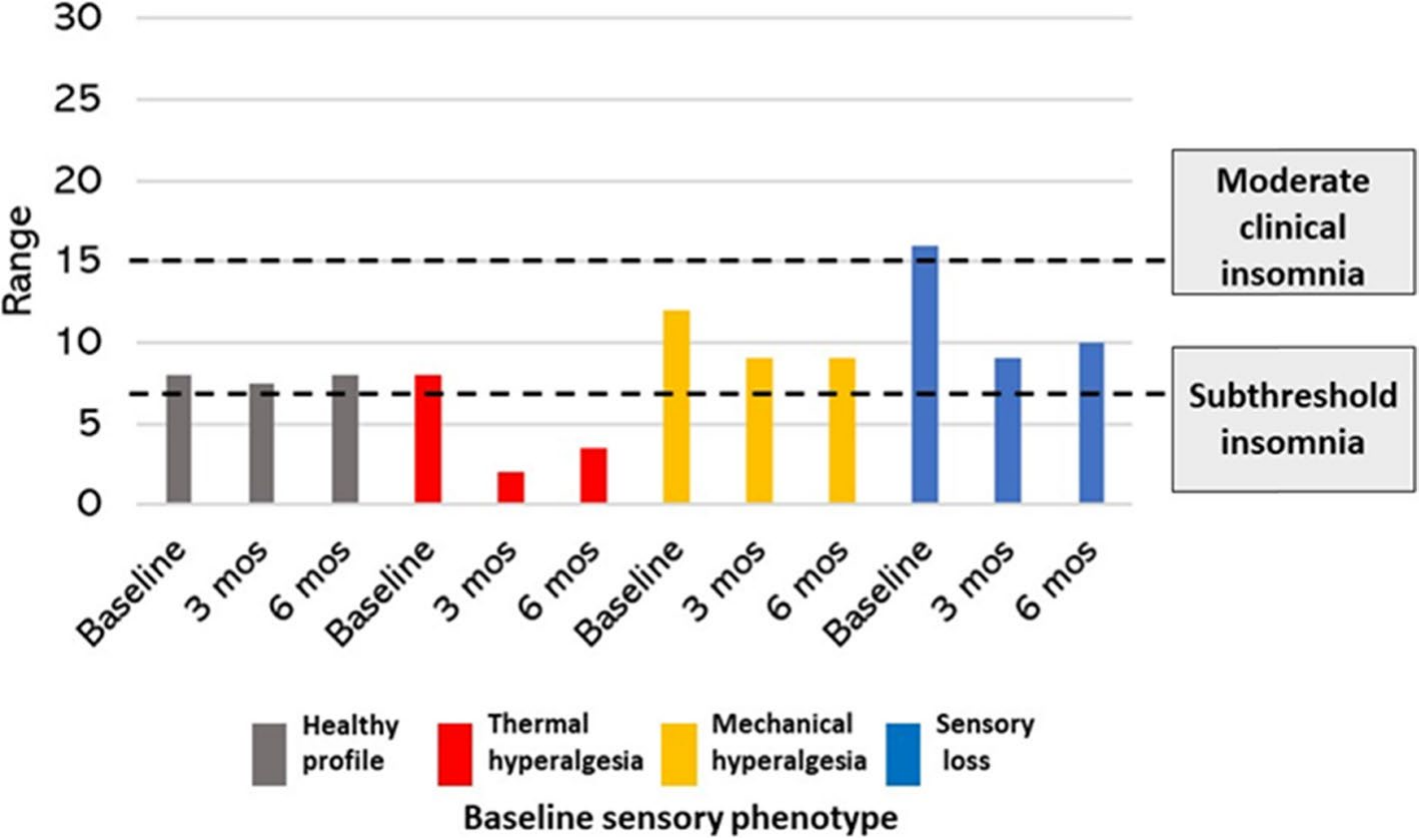
Median Insomnia Severity Index (ISI) score stratified by baseline sensory phenotype

Interrupted sleep is a hallmark of CTS and as anticipated, clinically relevant insomnia was prevalent in the sample. At baseline, scores for the Insomnia Severity Index (ISI) indicated 49(64%) participants had insomnia ranging from subthreshold to severe, with insomnia persisting in 31(46%) participants at 3 months post-surgery. Change in ISI scores across the 3 assessments was statistically significant; baseline [(median (IQR) 10.0 (11.25)]; 3-months post-surgery 5.5 (11.5), 6 months post-surgery 7 (11.5)] *P* < .001. Differences observed in the severity of sleep impairment, based on baseline sensory phenotype, were not statistically significant (*p* > .05) (Fig. 7).

### Functional interference

**Fig. 8:**
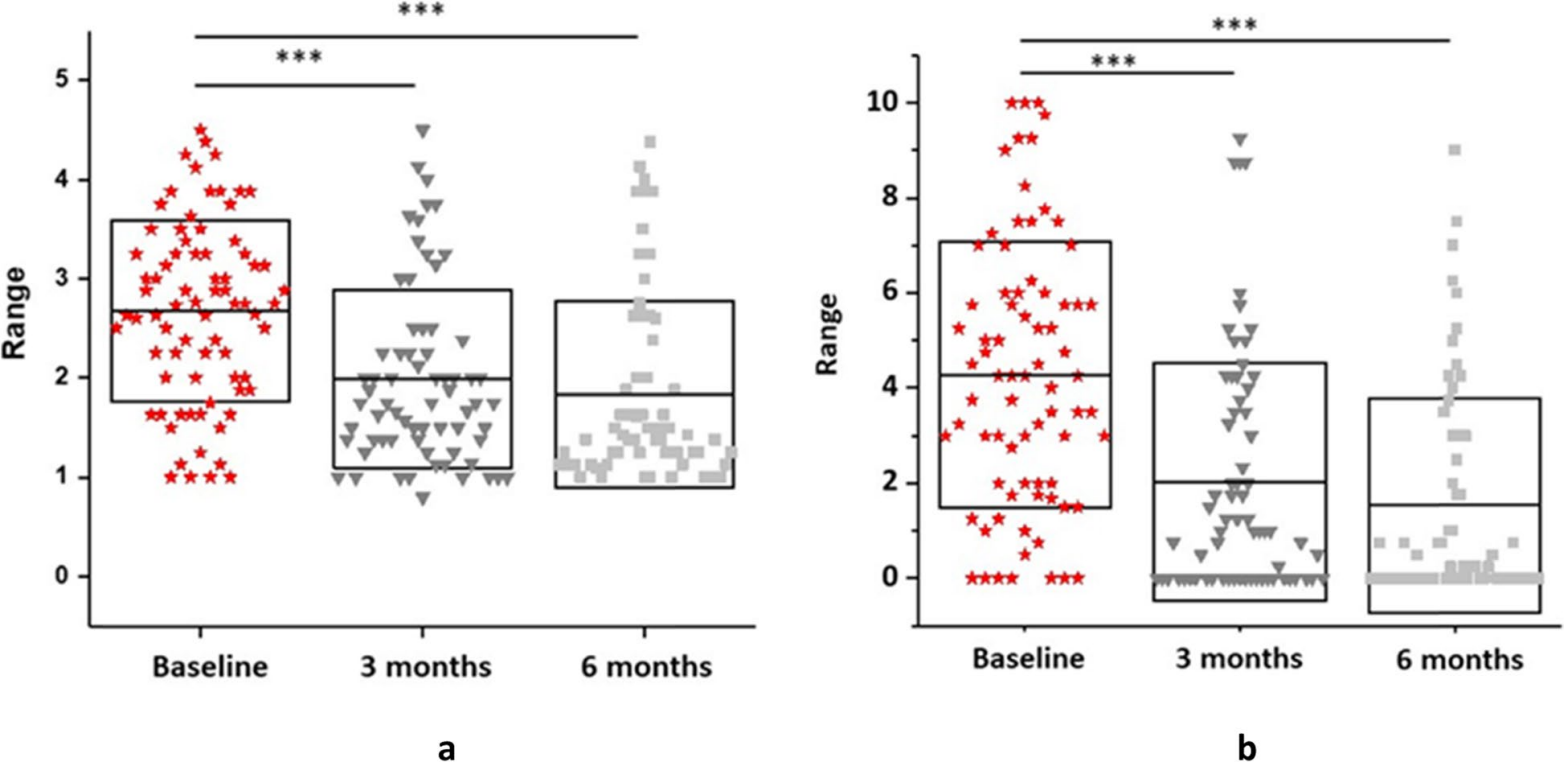
**a** Functional Status Scores (FSS). **b** BPI Pain Interference Scale. Change in functional status and pain interference scores across assessments*.* The grey boxes represent the standard deviation, centre line the mean at baseline, 3- and 6-months post-surgery. Each symbol represents a participant. Significance is denoted as * at the 0.05 probability level; ** at 0.01; *** at 0.001

Pain interference and functional impairment was disparate at baseline, with a large proportion of participants reporting moderate to severe dysfunction and interference. (Fig. 8a; 8b). For the sample, Functional Status Scores improved from baseline [mean (standard deviation) 2.64 (.85) to 3 months 1.96 (.89) and baseline to 6 months 1.8 (.9) with a large effect size (multivariate partial eta squared = .443) (Fig. 8a). Consistently, BPI pain interference scale scores improved from baseline [mean (SD) 3.64 (2.28)] to 3 months 1.52 (1.83)] and baseline to 6 months [1.61 (2.19)] with a large effect size (multivariate partial eta squared = .483) (Fig. 8b).

### Sensory phenotype associated burden

Pre-surgical health status, mood and functional deficit have been identified as potential prognostic factors for the outcome of carpal tunnel surgery. Therefore, we included these measures in an exploration of attendant burden related to QST derived sensory phenotype (Table 4). At baseline, CTS participants with mechanical hyperalgesia and sensory loss phenotypes have higher comorbidity scores, more severe sleep restriction and worse pain interference and functional impairment however these phenotypic differences did not reach statistical significance.

**Table 4:** Comparison of multimodal phenotyping measures between phenotypic groups at baseline

	Healthy Profile	Thermal Hyperalgesia	Mechanical Hyperalgesia	Sensory loss	*p* =
Comorbidity Score	3 (4)	4.5 (7)	5(7)	4(8)	.48
Depression	7 (5)	5 (2)	5.5 (6)	7 (4)	.27
Anxiety	4.5 (5)	3 (4)	5.5 (5)	4 (5)	.58
Insomnia Severity	8 (13)	8 (11)	12 (11)	16 (14)	.21
BPI Pain Interference	2.72 (3.57)	3.57 (3.61)	3.57 (4.22)	5.35 (3.72)	.15
Functional Status Score	2.32 (1.19)	2.75 (1.65)	2.76 (1.25)	3.00 (1.6)	.19

Data reported with median (interquartile range). Statistical significance reported for Kruskal-Wallis test. Baseline *N* = 76; healthy profile *n* = 16 (21%); thermal hyperalgesia *n* = 22 (29%); mechanical hyperalgesia *n* = 24 (32); sensory loss *n* = 14 (18%)

### Surgical outcome

**Fig. 9:**
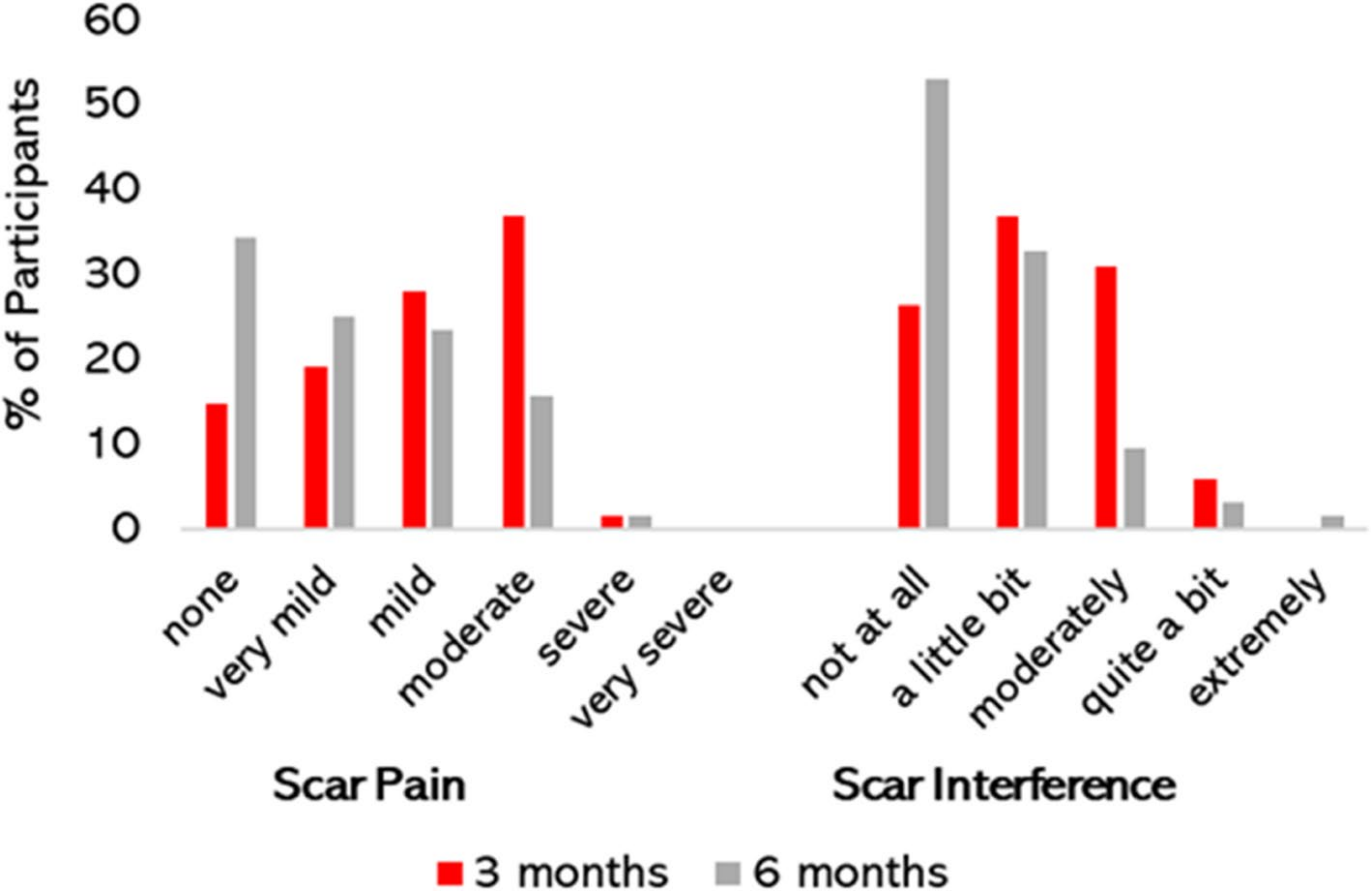
Patient-reported scar pain and interference. Frequency and severity of patient reported scar pain and functional interference at 3- and 6-months post-surgery

At 6 months post-surgery, 5 (8%) of participants reported a poor surgical outcome (worse or unchanged), 59 (92%) a good outcome (slightly better, much better or completely cured) (Table 5). At 3 months post-surgery 58 (85%) of participants reported scar pain which ranged from very mild to very severe (Fig. 9). Scar pain severity was diminished at 6 months however 42 (65%) of participants continued to report pain (very mild, 16 (25%); mild 15 (23%); moderate 10 (16%) and severe, 1 (1.6%). At 3 months post-surgery, 50 (74%) of participants reported some degree of scar interference, this was further reduced at 6 months with 30 (47%) participants reporting some degree of functional interference (Fig. 9).

**Table 5:** Patient-completed global rating of change at 3 and 6 months

Rating	3 months *n* = 68 n (%)	6 months *n* = 64 n (%)	
Worse	0	2 (3%)	poor outcome 8%
Unchanged	6 (9%)	3 (5%)	
Slightly better	11 (16%)	8 (13%)	good outcome 92%
Much better	41 (69%)	33 (52%)	
Completely cured	10 (15%)	18 (28%)

### Candidate prognostic outcome factors

Observed differences in pain, sleep impairment, psychological factors and function, between sensory phenotypic groups, was not significant. However, because sample size was predicated on one prognostic factor (difference in QST for a CTS cohort and controls), the study may have been under powered to detect between phenotypic group differences. The association of QST derived sensory phenotype, participant demographics and baseline multimodal phenotyping measures including pain parameters, psychological factors, sleep and functional impairment measures with patient global rating of change at 6 months post-surgery were investigated to identify potential factor candidates related to surgical outcome (Table 6). While differences in patient reported outcome between baseline sensory phenotypic groups were observed; a good surgical outcome was reported by 100% of those with a healthy sensory phenotype, 95% thermal hyperalgesia, 91% sensory loss and 85% mechanical hyperalgesia, these differences were not statistically significant (*p* = .51). Neither were the participant’s age, BMI, Comorbidity Score, duration of symptoms, nerve conduction study measured disease severity or pain parameters associated with surgical outcome (*p* > .05). However, the association of baseline psychological factors and functional interference with surgical outcome was statistically significant. The Pain Catastrophizing Scale score (*r* = −.248, *p* = .048); anxiety subscale of the DAPOS (*r* = −.366, *P* = .003) and BPI Interference scale (*r* = −.363, *p* = .003) were inversely correlated with patient reported surgical outcome; greater pain-related worry, greater anxiety and higher levels of functional interference were associated with a poorer surgical outcome.

**Table 6:** Correlation of baseline phenotyping parameters with patient reported global rating of change at 6 months

Patient global rating of change (PGRC)			
		*r* =	Sig. (2-tailed)
PAIN PARAMETERS	BPI pain severity score	−.185	.142
	DN4	−.227	.071
	Symptom Severity Score	−.136	.284
	NPSI	−.180	.154
PSYCHOLOGICAL MEASURES	Pain Catastrophizing Scale	**−.248**	**.048**
	Depression	−.221	.079
	Anxiety	**−.366**	**.003**
SLEEP	Insomnia severity index	−.115	.366
FUNCTION	Functional severity score	−.243	.053
	BPI pain interference scale	**−.363**	**.003**

Correlations interpreted as small *r* = .10 to .29; medium *r* = .30 to .49; large *r* = .50 to 1.0

*BPI* Brief Pain Inventory, *NPSI* Neuropathic Pain Symptom Inventory, *sig* statistical significance

## Discussion

In patients with chronically painful conditions, the large degree of inter-patient variability in treatment response has rationalized the need to identify measurable phenotypic characteristics of patients that are predictive of treatment outcomes [56]. This longitudinal study adopted such a multimodal phenotyping approach. To identify candidate prognostic factors, we explored the association of sensory phenotype and concomitant pain parameters, psychological factors, sleep restriction and functional impairment with outcome at 6 months post carpal tunnel surgery.

In this sample, a good surgical outcome was reported by 92% of participants. However, in this pragmatic cohort, a lenient cut-point of 3 (slightly better) was chosen a priori to determine surgical success. If a more stringent cut-point of grade of 4 (much better) was taken, then a good outcome would have been reported by 80% of participants and is more in keeping with the literature [7]. Our findings demonstrate that greater pain-related worrying, anxiety and functional interference, prior to surgery, are associated with poorer surgical outcome.

### Sensory phenotype and surgical outcome

In patients with peripheral neuropathic pain, the frequency of DFNS QST derived sensory phenotypes, thought to reflect different neurobiological mechanisms, differ between aetiologies [6]. In the current sample, at baseline, approximately 20% of participants presented with a healthy sensory profile, 30% with a thermal hyperalgesia phenotype, 30% with a mechanical hyperalgesia phenotype and approximately 20% with a sensory loss phenotype. This distribution of phenotypes is comparable to that reported in patients with peripheral nerve injury [6]. Observed differences in surgical outcome, between sensory phenotypic groups, were not significant. However, of clinical importance, while paraesthesiae and numbness are thought to be pathognomonic of carpal tunnel syndrome [2], our findings demonstrate that thermal and mechanical hyperalgesia sensory perturbations are observed as the dominant sensory feature in subgroups of this clinical cohort. Intriguingly, our findings demonstrate significant differences, between sensory phenotypic groups, in pain trajectory. At 6 months post-surgery, significant differences are observed for the Neuropathic Pain Severity Index whereby participants with mechanical hyperalgesia and sensory loss phenotypes demonstrate greater persisting neuropathic pain scores, perhaps supporting the hypothesis that sensory phenotype reflects different neurobiological mechanisms.

In this sample, prior to decompression surgery, loss of thermal detection (small fibre) and/or mechanical detection (large fibre) was more prevalent than gain to thermal or mechanical stimuli. Post-surgery, significant improvement is observed in large fibre function (vibration and mechanical detection), however at 6 months sensory function remains impaired compared to healthy controls, demonstrating recovering but persistent dysfunction. In contrast, a greater degree of small fibre function recovery is observed post-surgery. Cold detection threshold and thermal sensory limen improve significantly but remain reduced at 6 months, whereas warm detection threshold and cold pain threshold normalise at 3 months. These findings are consistent with the work of Baskozos et al. [75] who demonstrate with quantitative sensory testing, histologically and electrodiagnostically, that while improvement in large and small fibre encoded modalities is observed following median nerve decompression, recovery remains incomplete at 6 months. It is unclear, beyond 6 months, if large and small median nerve fibre function recovers further.

### Exploring pain in carpal tunnel syndrome

Findings for pain and symptom severity measures demonstrate that for patients with CTS, pain intensity, frequency and quality are heterogeneous experiences. At baseline 91% of participants reported pain of some severity (mean [standard deviation] pain severity score 4.17 [2.73]) however, of interest, pain parameters at baseline were not associated with patient rated surgical outcome. A significant improvement (reduction) for all pain parameters (DN4, NPSI, Symptom Severity Score and BPI pain severity score) was observed at 3 and 6 months post-surgery, consistent with findings in other carpal tunnel surgery cohorts [66, 76], however this does not imply pain resolution for all patients, as clearly illustrated.

In CTS, the mechanisms driving pain are not clearly elucidated and may change over the course of the disease. Theories based on the pathophysiology of nerve compression implicate ischemia secondary to raised intraneurial pressure, fibrosis and subsequent traction on a nerve that has been rendered immobile or possibly localised inflammatory processes beneath the transverse carpal ligament [26, 27]. It is intriguing that in CTS, patient-reported pain and symptom severity do not correlate with the severity of electrophysiologically assessed nerve conduction delay [14, 77, 78], in other words, more severe compression does not drive more severe pain.

Determining whether pain that is symptomatic of carpal tunnel syndrome is neuropathic in nature is important as this may have implications for treatment. Equally, it is important to understand the nature of persistent post-surgical pain in this group of patients for the same reason. Estimates of neuropathic pain in patients with carpal tunnel syndrome have ranged from 48 to 80% based on the tools used for diagnosis and sample characteristics [24, 77, 79]. In the current study, a stringent two-stage triage was used to define pain as neuropathic, or not [34] and 76% of participants were categorised as having neuropathic pain prior to surgery. Confirming the persistence of impaired median nerve somatosensory function, i.e., the persistence of a nerve lesion, post-operatively is essential in the diagnosis of post-operative neuropathic pain. In the current sample, at 6 months post-surgery, the incidence of neuropathic pain was found to be 12%, with 48% reporting pain that was non-neuropathic in nature and 40% reporting they were pain-free.

For many patients with CTS, pain control prior to surgery is not adequate. Furthermore, for a proportion of patients who undergo carpal tunnel decompression surgery, pain is not resolved. This unmet need has spurred a growing interest in the efficacy of vitamins and nutraceuticals for the reduction of pain and symptom severity in this patient population. Of particular interest, alpha-lipoic acid (ALA) is reported to exert an antioxidative, anti-inflammatory effect on peripheral nerves, thereby reducing sensory symptoms associated with peripheral neuropathy [80]. In patients with CTS, the administration of oral ALA both prior to and/or after surgery has been investigated in several blinded, randomised controlled trials with results suggesting some degree of efficacy in symptom and pain reduction [81–84]. While ALA appears a promising nutraceutical for the reduction of sensory symptoms in patients with CTS as well as improvement in carpal tunnel surgical outcomes, this evidence must be interpreted with some caution. Heterogeneity in study design including variability in ALA dose and frequency, disparity in the CTS symptom parameters of interest and the outcome evaluation of such and queries as to the adequacy of study sample sizes [82, 83] to detect between-groups differences suggest that further evidence of efficacy may be required.

### Pain-related worrying

Pain catastrophizing, or exaggerated, persistent thoughts related to painful experiences [67] has emerged as one of the most important psychological predictors of pain, distress, and disability [85]. However, a recent content analysis of pain catastrophizing self-report measures has raised questions as to the validity of assessing pain catastrophizing via self-report and suggests the construct being measured is more appropriately described as ‘pain-related worrying’ [69]. Simultaneously, a large international patient-researcher collaboration has rallied for a replacement term for pain catastrophizing in light of patient concerns that the term is pejorative, stigmatising and poses a barrier to care [86]. Therefore, the term pain-related worry has been used in the current work to describe the construct in question.

At baseline 33% of study participants were categorized as being high in pain-related worry based on their Pain Catastrophizing Scale (PCS) score. There was a significant decrease in scores up to 6 months, at which time 13% continued to report a high degree of pain-related worry. There is debate as to the nature of pain catastrophising as a construct, whether pain catastrophising is representative of a stable state or a situational trait [85, 87]. Our findings demonstrate a significant reduction in PCS scores (*p* < .001) with a parallel reduction or improvement in pain severity scores (<.001) from baseline to 6 months post-surgery, suggesting that when pain is effectively treated, catastrophic thought or pain-related worry diminishes.

In this sample, greater pain-related worry, prior to surgery, was found to be associated with poorer surgical outcome. This finding is supported by a recent prospective, observational study in 417 patients undergoing carpal tunnel decompression surgery; Mosegaard et al. [88] reported a statistically significant effect of preoperative Pain Catastrophizing Scale score on patient reported satisfaction.

### Anxiety

For three quarters of a century, preoperative anxiety has been recognized as a potentially modifiable risk factor for post-surgical complications [89, 90]. It is estimated that between 25 and 80% of patients admitted to hospital for surgery experience preoperative anxiety [91] and recognised that preoperative anxiety can adversely affect patient recovery [92]. In the current study, anxiety, as investigated with the DAPOS questionnaire, decreased significantly across all time points. Importantly, higher baseline anxiety scores were associated with poorer surgical outcome. This is consistent with findings from a large, multicentre cohort study of patients undergoing carpal tunnel surgery; a significant relationship was identified between anxiety and surgical outcome in this surgical cohort [23].

### Sleep disturbance

Surprisingly, in this sample of patients, Insomnia Severity Index (ISI) scores were not associated with patient-rated outcome. At baseline, ISI scores for 64% of participants suggested insomnia ranging from subthreshold to severe in intensity while at 6 months the frequency of insomnia was decreased to 46% of participants. In CTS, sleep interruption is a hallmark of the condition and is reported as a primary driver for patients to seek surgery. It is clear from the literature that the bidirectional relationship of sleep disturbance and pain is complex. A recent systematic review and meta-analysis in clinical populations reported that impaired sleep quality and quantity are associated with an increased risk of developing a chronic pain condition, small elevations in inflammatory markers and worse patient reported function [93]. There is also evidence from experimental studies that chronic insufficient sleep can alter pain modulation processes and induce sensitization, thereby increasing vulnerability to chronic pain [94, 95]. It is not clear, however, how or if these pain sensitisation processes normalise when sleep improves or normalises. It is possible that in the current cohort sleep impairment improved post-surgery but not of a sufficiency or length of time to impact on pain sensitivity, function and secondarily on patient-rated surgical outcome. It is also possible that sleep impairment was overestimated by participants both pre and post-surgery. Such a disparity between objective and subjective sleep measures is commonly reported in the literature [96] and is known to confound the evaluation of sleep and related impairment.

### Functional impairment

Reportedly, greater functional impairment prior to surgery, as evaluated with the Functional Status Scale (FSS) of the Boston Carpal Tunnel, is associated with poorer long-term outcome following carpal tunnel surgery [22]. In the present cohort of patients, the association of function as assessed with the FSS approached but did not reach significance (*p* = .053). However, a significant association was identified between baseline pain functional interference scores and patient reported global rating of change at 6 months post-surgery. The FSS comprises eight hand function specific items, including writing, fastening buttons and opening jars. In contrast, the pain interference scale of the Brief Pain Inventory (BPI) explores the degree to which pain interferes with seven areas of function, including general activity, mood, walking, work, interpersonal relationships, sleep and enjoyment of life [62]. The two measures therefore may be evaluating different constructs and considering function at different levels, with the FSS evaluating function at the impairment level and the BPI interference scale at the participation level.

### Scar pain and interference

At 6 months post- surgery 65% of CTS participants continued to report some degree of surgical scar pain and 47% reported scar interference. This finding is in keeping with the literature, scar pain and interference are reported to persist in some patients at up to 2 years post-surgery [97–99]. Despite evidence of the high incidence of problematic surgical scars following carpal tunnel surgery, patients are not routinely counselled prior to surgery as to this possibility of this adverse event and this is an important area for practice improvement.

### Study limitations

Study sample size was determined to enable the detection of a difference in somatosensory function (via quantitative sensory testing) between patients undergoing carpal tunnel surgery and healthy controls. Therefore, the study may have been underpowered to detect between-group differences in other phenotyping measures.

It was anticipated that up to 25% of participants enrolled in this study would report a poor surgical outcome, enabling the use of multivariate analysis of variance or regression analysis to robustly identify candidate prognostic factors. However, as only a small proportion of patients reported a poor outcome this was precluded; correlation was used to identify the association of phenotyping measures and surgical outcome.

At both study sites, surgery was performed by multiple surgeons. While the operating surgeon was not included as an outcome variable, patient-reported surgical outcome was consistent with that reported in the literature.

All patients scheduled for carpal tunnel surgery were invited to participate, however it is impossible to control for participant-selection bias. In clinical studies of this nature, the clinician-patient interaction cannot be ruled out and may influence or bias the patients’ perception or judgment of outcome. To reduce the likelihood of assessor bias the investigator (DLK) was blinded to patient reported results of surgical outcome until patients completed the trial. The completed measures were placed in pre-labeled, coded envelopes by the study participants and secured in the written case report form. As the baseline and post-surgical measures were completed by one investigator in this study, one cannot rule out the risk of investigator bias.

Study findings may not be generalizable to other care pathways, for example in settings where there is minimal waiting time from patient presentation to surgery.

## Conclusions

This study explored the association of QST derived sensory phenotype and associated comorbid burden with the outcome of carpal tunnel surgery with the aim of identifying candidate prognostic outcome factors for future investigation. Our findings demonstrate that patients with CTS present with significant heterogeneity in somatosensory function; while post-surgical recovery is observed, dysfunction persists in both small and large fibre function at 6 months. Differences between sensory phenotypic groups in pain parameters, pain catastrophising, mood, insomnia and functional impairment were observed, suggesting greater burden in patients with a mechanical hyperalgesia or sensory loss phenotype, however these differences did not reach statistical significance in this sample size. Similarly, phenotypic group differences in surgical outcome were observed but did not reach statistical significance. In patients with CTS, pain-related worry is prevalent but improves significantly post-surgery with a concomitant reduction in pain. Sleep impairment, assumed secondary to symptom severity, persists in a large proportion of patients post-surgery, despite symptom reduction. Greater pain-related worrying, anxiety and pain interference prior to surgery, are associated with poorer surgical outcome. These candidate prognostic outcome factors require further investigation in prognostic factor modelling studies.

### Clinical implications

- There is a growing body of evidence that pain-related worry and anxiety are associated with the outcome of carpal tunnel surgery. At present, pre-surgical clinical assessment does not routinely include the evaluation of known psychological risk factors. Where high levels of anxiety are identified, cognitive behavioural therapy (CBT) has been shown to be effective [100] and warrants consideration for implementation in surgery “prehabilitation” programmes. In addition, patient codesign of surgical care pathways might be explored, to ensure that pathways are patient-centred to the greatest degree possible.
- Prior to carpal tunnel surgery, patients should be informed as to the possibility of prolonged or persistent scar discomfort and interference. Scar outcomes have important implications particularly so for those returning to manual work.
- Consistent with previous reports [101], patients with carpal tunnel syndrome do not present with dynamic mechanical allodynia, neither prior to nor following decompression surgery. Allodynia is frequently observed in patients with complex regional pain syndrome (CRPS) [28], a severe, debilitating chronic pain condition which is known to occur, albeit rarely, as a potential severe complication of carpal tunnel decompression surgery [102]. Allodynia is not pathognomonic of CRPS, however where detected in patients with CTS or following carpal tunnel surgery investigation to rule out CRPS is warranted.

## Data Availability

Healthy volunteer quantitative sensory testing data is available at 10.6084/m9.figshare.12860066.v2. Additional datasets used and/or analysed during the current study are available from the corresponding author on reasonable request.

## Abbreviations

ANOVA: Analysis of variance
BCTQ: Boston Carpal Tunnel Questionnaire
BPI: Brief Pain Inventory
BPI-SF: Brief Pain Inventory Short Form
CTR: Carpal tunnel release
CTS: Carpal tunnel syndrome
CBT: Cognitive behavioural therapy
CDT: COLD detection threshold
CPT: Cold pain threshold
CRPS: Complex regional pain syndrome
DAPOS: Depression, Anxiety and Positive Outlook Scale
DN4: Douleur Neuropathique 4 questions
FSS: Functional Status Scale
DFNS: German Research Network on Neuropathic Pain
HPT: Heat pain threshold
*IMMPACT*: Initiative on Methods, Measurement, and Pain Assessment in Clinical Trials
ISI: Insomnia Severity Index
MDT: Mechanical detection threshold
MPS: Mechanical pain sensitivity
MPT: Mechanical pain threshold
NHS: National Health Service
NCS: Nerve conduction studies
NPSI: Neuropathic Pain Symptom Inventory
PCS: Pain Catastrophizing Scale
PSS: Pain Severity Score
PGRC: Patient-reported global rating of change
PPT: Pressure pain threshold
QST: Quantitative sensory testing
SSS: Symptom Severity Scale
TSL: Thermal sensory limen
VDT: Vibration detection threshold
WDT: Warm detection threshold
WUR: Wind-up ration
